# Investigation and development of natural products that target chemotherapy resistance factors in cancer cells

**DOI:** 10.1007/s11418-025-01942-2

**Published:** 2025-08-08

**Authors:** Takahiro Matsumoto

**Affiliations:** Division of Biological Sciences-Laboratory of Public Health, Kyoto Pharmaceutical University, Yamashina-Ku, Misasagi, Kyoto 607-8412 Japan

**Keywords:** Cancer stem cell, Heat shock protein, Wnt/*β*-catenin pathway, Chemotherapy resistance factor

## Abstract

Heat shock proteins (HSPs) play an important role in several tumors; contribute to anti-cancer drug resistance, cell proliferation, and metastasis; and have been suggested as a major cause of failed anti-cancer drug treatment. In addition, cancer stem cells (CSCs) have been identified in many types of malignancies, including leukemia, breast, colorectal, and brain cancers, and are a leading cause of failed cancer treatment owing to their anti-cancer drug and radiation therapy resistance. Therefore, many researchers, including our group, have investigated and developed natural products that target chemotherapy resistance factors in cancer cells. This review introduces the inhibitors of chemotherapy resistance factors discovered using a unique assay system.

**Graphical Abstract:**

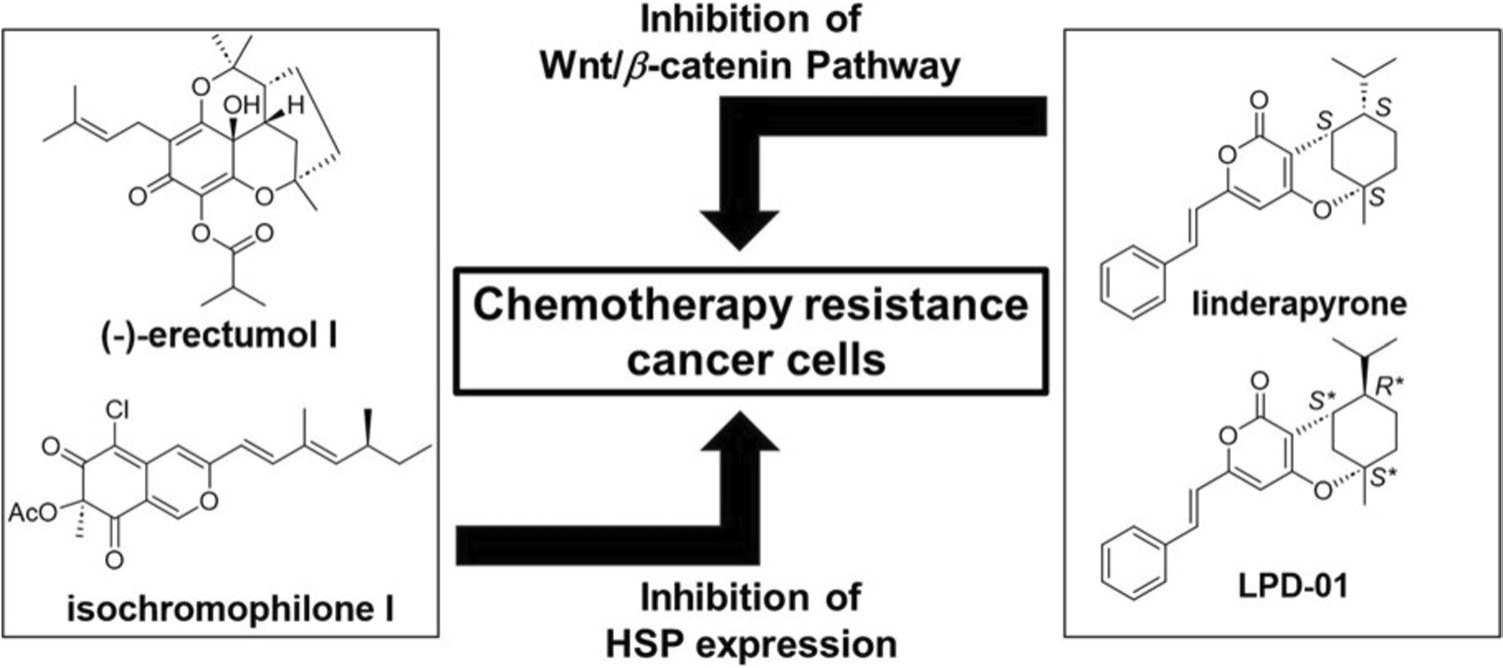

## Introduction

As a result of the long-term and broad research aimed at discovering new anti-cancer drugs from plants, marine organisms, and microorganisms, several types of anti-mutagenic drugs have been reported to exhibit significant activity. These compounds have contributed to cancer treatment for a long time; however, drug resistance occurs through the anti-apoptotic functions of heat shock proteins (HSPs) overexpressed in several cancer cells [1], the occurrence of cancer stem cells (CSCs) resistant to current anti-cancer drugs [2, 3], activation of DNA repair pathways [4], mutation of treatment targets [5], activation of detoxifying enzymes [6], and expression of the drug efflux transporter P-glycoprotein (P-gp) [7]. Therefore, these drug resistance factors are important targets for new drug discoveries to avoid cancer recurrence.

For example, adriamycin (ADR) has been used as a potent chemotherapeutic drug for the treatment of several cancers [8]. ADR induces cell death by intercalating into the base pairs of the DNA double helix. Although ADR is effective in tumor therapy, its utility is limited owing to its side effects, including cardiotoxicity [9]. In addition, many types of cancer cells highly express HSPs, which are major factors involved in ADR resistance [10]. A recent study revealed that decreased HSP105 expression enhanced apoptosis induction in ADR-treated HeLa cells [1]. Therefore, compounds that inhibit HSP expression may help prevent recurrence after tumor therapy.

Next, CSCs have been identified in many types of malignancies, including leukemia, breast, colorectal, and brain cancers, and are a leading cause of failed anti-cancer drug treatment. These cells are resistant to current anti-cancer drugs and radiation therapy and play important roles in metastasis by acquiring mesenchymal properties, including enhanced motility and invasive ability. Additionally, CSCs can self-renew and become tumorigenic [11, 12]. Therefore, compounds cytotoxic to CSCs could be used as therapeutic agents against cancer.

Therefore, many researchers, including our group, have investigated and developed natural products that target chemotherapy resistance factors in cancer cells. In this review, I highlight the inhibitors of HSP expression [13–15] and CSC-related cell signaling pathways [16–26], which may have potential as new seed compounds for cancer treatment.

### Evaluation of the cell death-inducing activity against ADR‑treated HeLa cells via time-lapse imaging

**Fig. 1:**
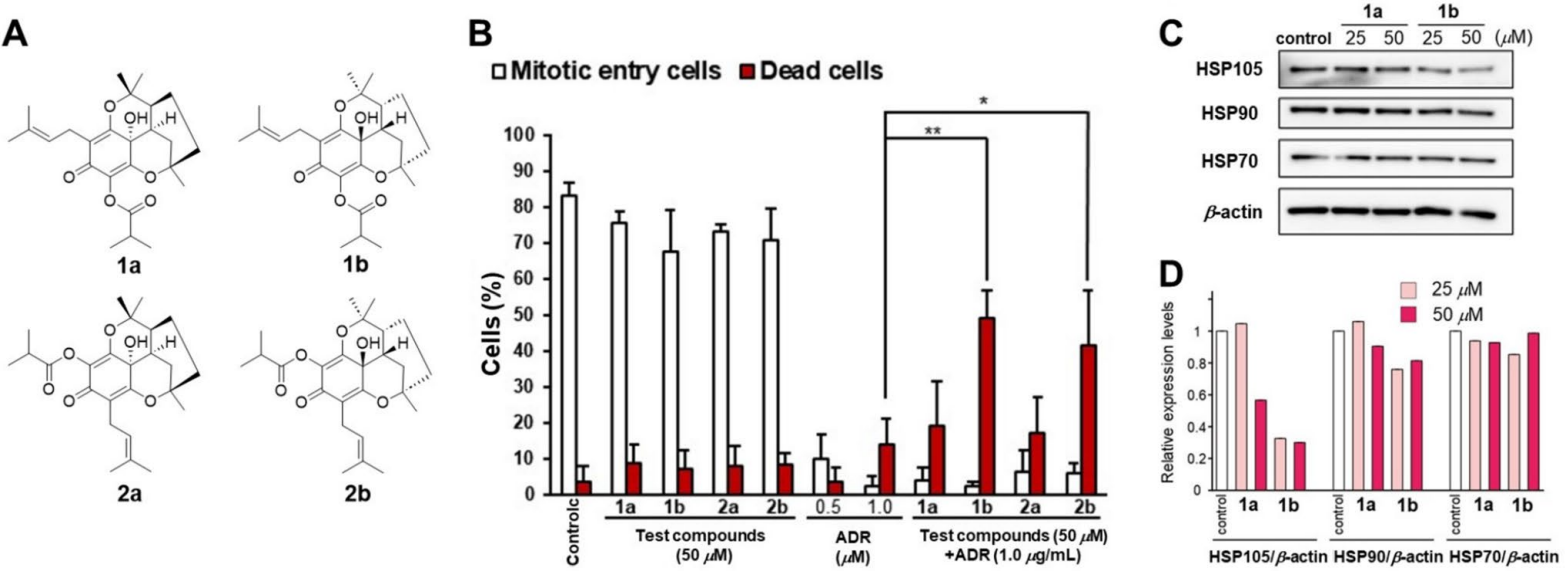
Effects of *Hypericum erectum* constituents on cell proliferation, cell death, and heat shock protein (HSP) expression in HeLa cells. **A** Chemical structures of constituents (**1a**, **1b**, **2a**, and **2b**) isolated from *H. erectum*. **B** HeLa cells were treated for 24 h with 50 μM of the indicated compounds, ADR (0.5 and 1.0 μg/ml), or a combination of both. The number of cells that entered mitosis and the number of dead cells were counted by time-lapse imaging. The results are reported as mean ± standard deviation of triplicate determinations. Statistical significance was analyzed using the Tukey–Kramer test (*P < 0.01 and **P < 0.001 compared with 1.0 μg/ml ADR-treated cells). **C** Western blots of HSP105, 90, and 70 expression levels in HeLa cells treated with **1a** and **1b** for 24 h. (D) Bar graphs of the relative expression levels of HSPs/*β*-actin compared to those of control in the western blots using ImageJ software

ADR inhibits cell proliferation by inducing G_2_/M cell cycle arrest. A previous study suggested that the cell cycle arrest was induced by DNA damage, but that low concentrations of ADR (0.1–1.0 μg/ml) do not induce cell death [1, 27]. Therefore, compounds that increase the number of dead cells after treatment with low concentrations of ADR may reduce the required dosage of ADR, avoiding associated side effects in tumor therapy. We evaluated the cell death-inducing activities of naturally occurring compounds isolated from medicinal plants against ADR-treated HeLa cells over 24 h using time-lapse imaging analysis [14]. In this study, we counted the number of mitotic entry and dead cells after treatment with a low ADR concentration (1.0 μg/ml), the compounds of our natural product liberally (test compounds), and a combination of test compounds and ADR (1.0 μg/ml). Among the naturally occurring compounds, prenylated phloroglucinol derivatives ( −)-erectumol I **(1b**) and ( +)-erectumol II **(2b**) (Fig. 1A), isolated as new compounds from the methanol extracts of whole *Hypericum erectum* plants, showed significant cell death-inducing activity against ADR-treated HeLa cells (Fig. 1B).

( −)-Erectumol I **(1b**) and ( +)-erectumol II **(2b**) were isolated with other novel compounds, ( +)-erectumol I (**1a**) and ( −)-erectumol II (**2a**). These compounds were isolated as pairs of enantiomers (Fig. 1A). Their planar chemical structures and relative configurations were determined by Cu-Kα X-ray diffraction analysis and confirmed by high-resolution mass spectrometry and 1D and 2D NMR spectroscopy. The absolute configurations of the four new compounds were established by comparing the experimental and predicted electronic circular dichroism (ECD) data. Treatment with **1a, 1b, 2a,** and **2b** alone (50 μM) did not affect the number of dead cells and mitotic entry cells. On the other hand, the combined treatment of **1b** or **2b** (50 μM) with ADR (1.0 μg/mL) significantly increased the number of dead cells compared to treatment with ADR alone (1.0 μg/mL) (Fig. 1B). These results suggest that **1b** and **2b** may be potent anti-cancer agents that enhance the efficacy of several cancer drugs. Interestingly, enantiomers **1a** and **2a** did not increase the number of dead cells compared to that among ADR-treated cells. Therefore, the targets of **1b** and **2b** should have chiral centers and may be key proteins with anti-apoptotic functions in cancer cells [14].

### Inhibitory effects of 1a, 1b, 2a, and 2b on HSP expression levels in HeLa cells

Many types of cancer cells highly express HSPs, which are major factors involved in ADR resistance [10]. I would like to introduce the previously reported inhibitors of several types of HSPs. HSP90 has been a target for cancer treatment since the 1990s, and various natural compounds with inhibitory activities have been reported. The first reported HSP90 inhibitor was geldanamycin, produced by *Streptomyces*, which inhibits HSP90 function by binding to its N-terminal ATP-binding site [28]. Furthermore, geldanamycin derivatives with improved water solubility and reduced hepatotoxicity have undergone their first clinical trials as HSP90 inhibitors. However, these derivatives did not achieve sufficient efficacy and were not put into practical use. On the other hand, research on geldanamycin and its derivatives has shown that compounds with affinity for the N-terminal ATP-binding site of HSP90 effectively inhibit its function. Numerous inhibitors have been developed based on geldanamycin and the partial structures of ATP. For instance, pimitespib was approved in Japan in June 2022 for the treatment of gastrointestinal stromal tumors that worsened after cancer chemotherapy [29, 30].

However, HSP90 inhibition reportedly induces other HSPs, such as HSP70 [31, 32]. Several natural HSP70 inhibitors have been reported, including the plant-derived lapachol and coelenterate-derived malonganenone A [33]. These compounds were identified using a protein aggregation assay, and their inhibitory effects were confirmed by assessing the molecular chaperone function of HSPs. HSP105 is one of the molecular chaperones that suppresses ADR-induced cell death by inhibiting apoptosis. In addition, HSP105 is overexpressed in several types of cancer cells, including HeLa cells; therefore, HSP105 inhibition has been suggested as a new target for cancer chemotherapy. Small interfering RNA (siRNA) knockdown of HSP105 in HeLa cells enhanced ADR-induced cell death [1]. However, no publications have described naturally occurring HSP105 inhibitors, other than our report on guaiane-type sesquiterpene kessyl glycol diacetate [13].

Based on the above knowledge and results from the evaluation of the cell death-inducing activity in ADR-treated HeLa cells via time-lapse imaging, we hypothesized that **1b** and **2b** may inhibit HSP function or expression. Therefore, we evaluated the expression levels of HSP105, 90, and 70 in HeLa cells treated with **1a** and **1b** by western blot analysis. We selected **1a** and **1b** for this analysis because we wanted to compare the relationship between the results of time-lapse imaging and western blot analysis. Treatment with 25 μM **1b** inhibited HSP105 expression without affecting HSP70 or HSP90 expression. At 50 μM, both **1a** and **1b** inhibited HSP105 expression (Fig. 1C and D). These results corresponded to the increased death of ADR-treated HeLa cells. Therefore, we concluded that a mechanism of cell death induced by **1b** was inhibited HSP105 expression [14].

### Evaluation of the inhibitory effects of natural products against HSP105 expression via a luciferase assay system

**Fig. 2:**
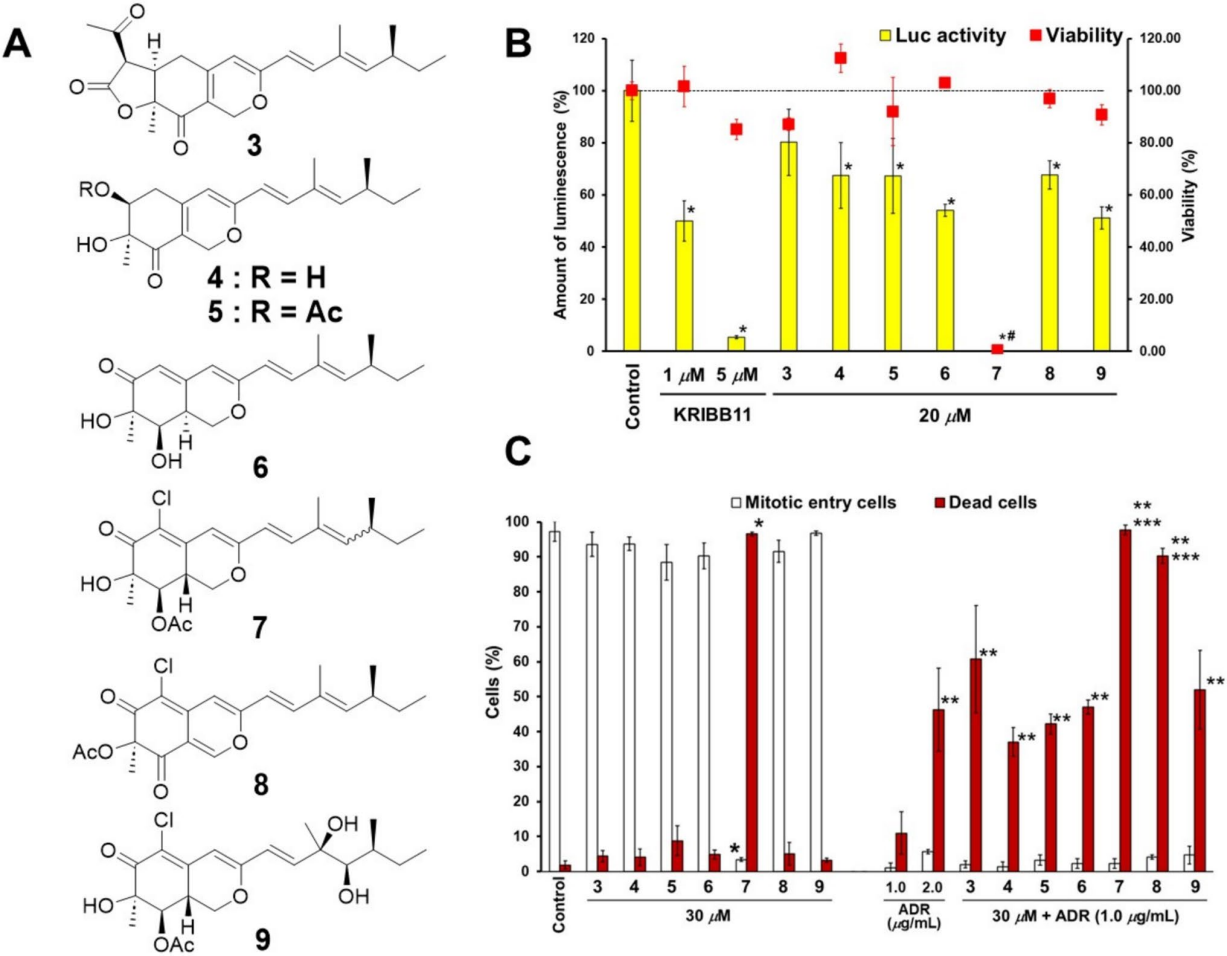
Effects of compounds produced by *Penicillium maximae* JKYM-AK1 on heat shock protein promoter activity in pGL105/C3H cells and on cell proliferation and death in HeLa cells. **A** Chemical structures of compounds (**3**–**9**) produced by *P. maximae* JKYM-AK1. **B** Inhibitory effects of **3**–**9** on heat shock protein promoter activity as determined using a luciferase assay system with pGL105/C3H cells. The percentages of luciferase activities and cell viabilities are described as means ± SD (*n* = 3) from three independent experiments. Statistical significance was analyzed using Dunnett’s test (*P < 0.01, ^#^*P* < 0.01 compared with each control group). **C** Effects of **3**–**9** on cell proliferation and death. The numbers of mitotic entry and dead cells were counted during time-lapse imaging. The percentages of mitotic entry and dead cells are reported as means ± SD of three different visual fields. More than 100 cells were captured in each field. Statistical significance was analyzed using the Tukey–Kramer test [*P < 0.01 compared with the control group, **P < 0.01 compared with the ADR (1.0 mg/ml) group, ***P < 0.01 compared with the ADR (2.0 mg/ml) group.]

As described in the above section, we succeeded in finding HSP105 inhibitors using a time-lapse imaging system with ADR-treated HeLa cells; however, a more efficient screening system to identify HSP105 expression inhibitors is desirable. Therefore, we evaluated the inhibitory effects of natural products on HSP105 expression with a luciferase (*luc*) assay system using pGL105/C3H cells. The pGL105/C3H cells were mouse C3H10T1/2 cells stably transfected with the pGL105 reporter plasmid containing the HSP105 promoter upstream of the luciferase gene. In this assay, the inhibitory effects of the test samples were assessed by observing a decrease in *luc* activity [34]. KRIBB11 was used as the positive control. KRIBB11 has been reported to inhibit heat shock factor 1 (HSF-1), a transcription factor for several HSPs, including HSP105 [35]. Using this assay, we found that azaphilones (Fig. 2A) isolated from the mycelia and supernatant after incubation with the airborne-derived fungus *Penicillium maximae* JKYM-AK1 significantly inhibited *hsp105* promoter activity without cytotoxicity (Fig. 2B).

Azaphilones (**3**–**9**), including four novel compounds, maximazaphilones I–IV (**3**–**6**), and three known compounds, isochromophilone IV (**7**), isochromophilone I (**8**), and hypocrellone A (**9**), were isolated. The chemical structures of the newly isolated compounds were elucidated based on chemical and physicochemical evidence, such as NMR and MS spectra. For maximazaphilones I, II, and IV (**3**, **4**, and **6**), the absolute configurations were established by comparing the experimental and predicted ECD data. Among these compounds, **4**–**9** significantly decreased *luc* activity; however, **7** significantly inhibited cell proliferation (Fig. 2B). These results suggest that compounds **4**–**6**, **8**, and **9** inhibit HSP105 expression in pGL105/C3H cells. We could not determine whether the inhibitory effect on *luc* activity was caused by the cytotoxicity of **7** [15]. 

### Evaluation of the cell death-inducing activity and inhibitory effects of compounds 3–9 against HSP expression

We evaluated the cell death-inducing activity of compounds (**3**–**9**) against HeLa cells for 24 h using time-lapse imaging. Treatment with compounds **3**–**6**, **8**, and **9** did not affect the number of dead or mitotic entry cells. In contrast, **7** increased the number of dead cells and decreased the number of cells entering mitosis, similar to the results of the *luc* assay using pGL105/C3H cells. The combination treatment of all isolated compounds (**3**–**9**) with ADR (1.0 μg/ml) significantly increased the number of dead cells compared with that among cells treated with ADR alone (1.0 μg/ml) (Fig. 2C). Therefore, **3**–**6**, **8**, and **9** may suppress the anti-apoptotic functions of HeLa cells. Moreover, the number of dead cells after the combination treatment of **8** with ADR (1.0 μg/ml) was significantly higher than that in the ADR (2.0 μg/ml) treatment group. These results suggested that **8** treatment may allow a less than 50% dose of ADR, which may reduce side effects. According to the results of the *luc* assay and cell death-inducing activity, **8** may have inhibitory effects on HSP105 expression. Therefore, we evaluated the expression of HSP105 and major HSPs, HSP90 and HSP70, in HeLa cells treated with **8** using western blot analysis. Treatment with **8** (100 μM) inhibited HSP105 and HSP90 expression under heat shock conditions; however, **8** (30 μM) had no effect. Therefore, we concluded that the mechanisms of the cell death-inducing activity of **8** may inhibit not only HSP105 and HSP90 expression, but also other effects contributing to drug resistance [15].

### Evaluation of TCF/β-catenin transcriptional activity for discovery of Wnt/*β*-catenin pathway inhibitors

**Fig. 3:**
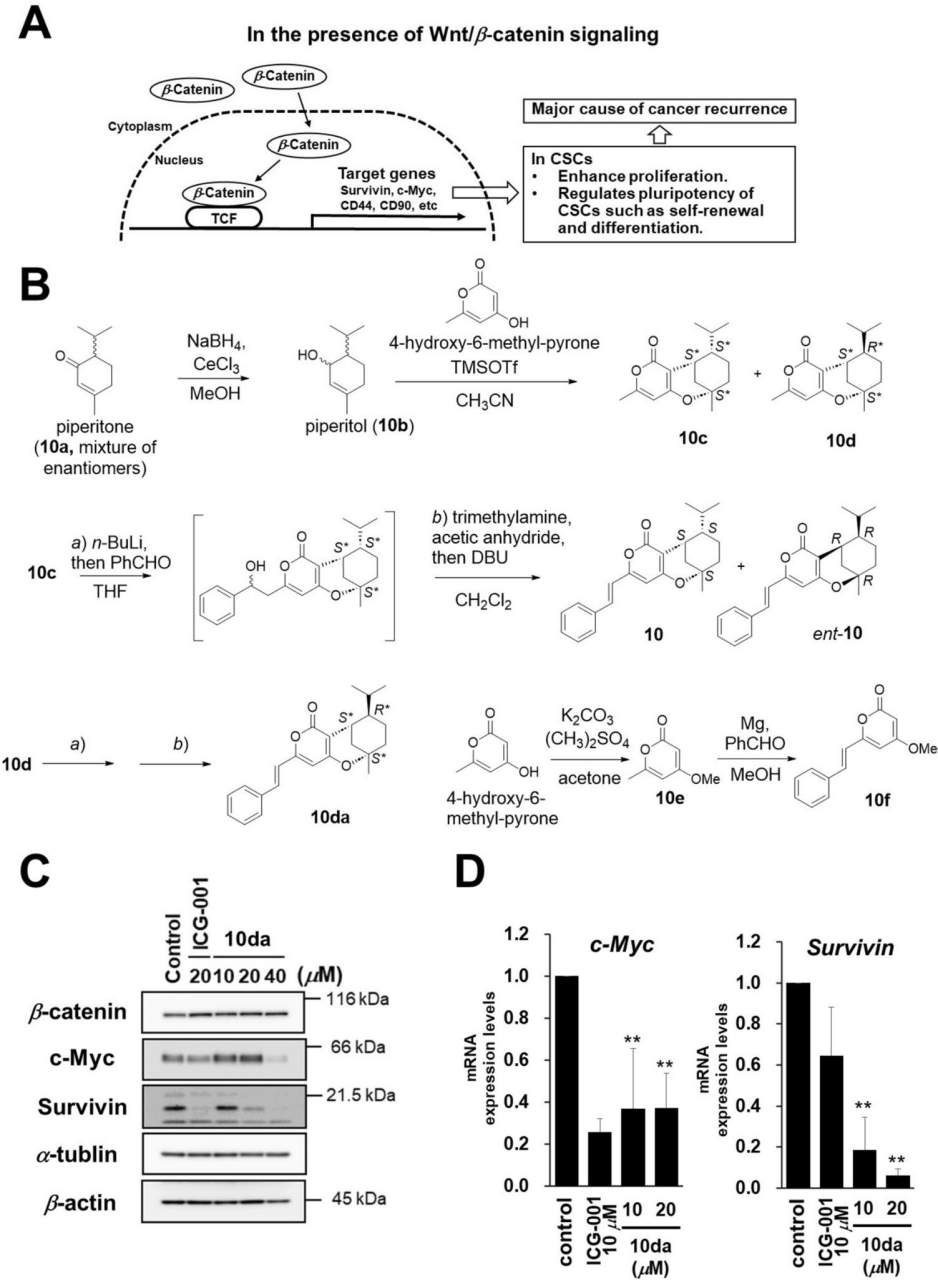
The role of Wnt/*β*-catenin pathway on CSCs, synthesis of **10**, **10da**, and related compounds together with the expression levels of mediator and target genes of the Wnt/*β*-catenin signaling pathway in HT-29 cells treated with **10da**. **A** In the absence of Wnt signaling, serine phosphorylation of *β*-catenin by the Axin–glycogen synthase kinase–3*β* complex targets *β*-catenin for ubiquitination degradation. In the presence of Wnt signaling, the activity of the Axin complex is inhibited and *β*-catenin enter the nucleus. Then, *β*-catenin binds to TCF to form a complex, which enhances gene expression that related to pluripotency of CSCs. **B** Synthesis of linderapyrone (**10**) and related compounds for the structure–activity relationship study. **C** HT-29 cells were treated with the indicated concentrations of the compounds for 24 h. Cell lysates were collected, and the Wnt/*β*-catenin signaling-related proteins were detected by western blotting. **D** HT-29 cells were treated with the indicated concentrations of compounds for 24 h. Quantitative reverse transcription PCR (RT-qPCR) was performed to evaluate *c-myc* and *survivin* gene expression levels. The amount of *β*-actin mRNA was used to normalize the data. Data are presented as mean ± SD of three independent experiments (*P < 0.05, **P < 0.01, compared with the control)

CSCs are resistant to current anti-cancer drugs and radiation therapy and play important roles in metastasis by acquiring mesenchymal properties. Therefore, CSCs are attractive targets for developing new anti-cancer therapies and the combination of conventional anti-cancer therapies with drugs targeting CSC-related pathway could lead to cancer eradication. The Wnt/*β*-catenin pathway plays one of the most important roles in the proliferation of CSCs (Fig. 3A) [36, 37]. For instance, the activation of Wnt/*β*-catenin pathway induces the transformation of dormant CSCs into active CSCs to promote cell cycle progression via increasing the expression of downstream cyclin D1 and MYC [38]. In addition, aberrant Wnt signaling has been observed in the self-renewal of CSCs. Indeed, capillary morphogenesis gene 2 induces nucleus accumulation of β-catenin to regulate the self-renewal and tumorigenicity of gastric CSCs [39]. Wnt signaling also plays an important role in the dedifferentiation of CSCs. The Lgr5, a member of the G protein-coupled receptor family that is located downstream of the Wnt signaling pathway, restrains the differentiation of esophageal squamous cell carcinoma stem cells [40]. Therefore, we focused on Wnt/*β*-catenin pathway inhibitors to discover cancer treatment agents. To find new Wnt/*β*-catenin pathway inhibitors, we evaluated the inhibitory effects of several natural products on TCF/*β*-catenin transcriptional activity (TOP activity) using STF/293 cells [17]. The human embryonic kidney 293 cells were stably transfected with modified M50 Super 8 × TOPFlash (*luc* reporter plasmid containing luciferase gene at downstream of the TCF-binding site) with a hygromycin resistance gene obtained from the pGL4.32 vector as reported previously [41–43]. In this assay, the inhibitory effects of the test samples were assessed by observing changes in *luc* activity [44, 45]. As a result of our study using our natural product liberally, the novel compound linderapyrone (**10**), isolated from *Lindera umbellata*, exerted a significant inhibitory effect without cytotoxicity (Fig. 3B) [17].

The chemical structure of linderapyrone (**10**), including its absolute stereochemistry, was elucidated based on chemical and physicochemical evidence, such as NMR, MS, and ECD data. To evaluate the structure–activity relationship between **10** related compounds and TOP activity, we synthesized** 1** from piperitone (**1a**) according to a previous report (Fig. 3B). Catalytic reduction of piperitone **(10a**, a mixture of enantiomers) yielded piperitol (**10b**). Through acid-promoted S_N_1 substitution and intramolecular etherification, **10c** (minor product) and **10d** (major product) were obtained from **10b**. Finally, **10c** was treated with *n*-BuLi at −78 °C and benzaldehyde to obtain a reaction intermediate that was treated with Ac_2_O/triethylamine, followed by the addition of 1,8-diazabicyclo[5.4.0]undec-7-ene to obtain ( ±)-**10** (mixture of **10** and its enantiomer). HPLC separation on a chiral column afforded optically pure** 10** and its enantiomer (***ent-10***). Using the same reaction conditions, **10da** (a diastereomer of **10**, racemic mixture) was synthesized from** 10d**. In addition, 4-methoxy-6-methylpyrone (**1e**) and 5,6-degydrokawain (**1f**) were synthesized for structure–activity relationship studies.

The inhibitory effects against TOP activity of piperitone (**10a**), its synthetic intermediates (**10c** and **10d**), and related compounds (*ent*-**10**, **10d**, **10e**, and **10f**) were evaluated. The enantiomer *ent*-**1** and diastereomer **1da** showed significant inhibitory effects, and their effects were equivalent to those of the natural product **10**, whereas monoterpene **10a** and pyrones **10e** and **10f** showed no inhibitory effects. In contrast, the synthetic intermediates **10c** and **10d** with both moieties showed weak effects. Based on these data, some structure–activity relationships have been suggested. Namely, the stereochemistry of the monoterpene moiety does not limit the inhibitory effects of 10 and its related compounds against TOP activity. Both monoterpene and pyrone moieties are necessary, and the phenyl group at C-8 may contribute to the enhancement of these effects [17].

### Evaluation of the inhibitory effects of 10, *ent*-10, and 10da against the Wnt/*β*-catenin pathway in HT-29 human colon cancer cells

The inhibitory effects of **10**, ***ent-10***, and **10da** against TOP activity were confirmed based on the viability of HT-29 human colon cancer cells. The Wnt/*β*-catenin pathway contributes to HT-29 cell proliferation. The viability of HT-29 cells significantly decreased after **10**, ± **10**, and **10da** treatment for 72 h. The IC_50_ values of **10** (IC_50_:32.4 ± 2.3) and ± **10** (IC_50_:19.9 ± 7.6) were not stronger than those of the positive controls ICG-001 (IC_50_:10.8 ± 2.0) and IWR-1 (IC_50_:9.52 ± 1.2). In contrast, **10da** (IC_50_:8.2 ± 2.3) exhibited cytotoxicity at lower concentrations than did the positive controls.

Based on these results, we hypothesized that **10da** has potential as a new seed compound for colon cancer treatment and named it LPD-01 (**10da**) in further mechanistic studies. Namely, we examined the expression levels of the transcriptional co-activator (*β*-catenin) and target proteins (c-myc and survivin) of this signaling pathway in **10da**-treated HT-29 cells using western blotting. The results showed that **10da** decreased c-myc and survivin expression levels, but not *β*-catenin levels (Fig. 3C). In addition, *c-myc* and *survivin* mRNA expression were downregulated in HT-29 cells (Fig. 3D). These results suggested that the inhibition of the Wnt/*β*-catenin signaling pathway by **10da** was not due to *β*-catenin degradation but to other mechanisms, such as the inhibition of *β*-catenin nuclear translocation or *β*-catenin/TCF complex formation [25].

### Identification of the target protein of the novel Wnt/*β*-catenin pathway inhibitor 10da on HT-29 cells

**Fig. 4:**
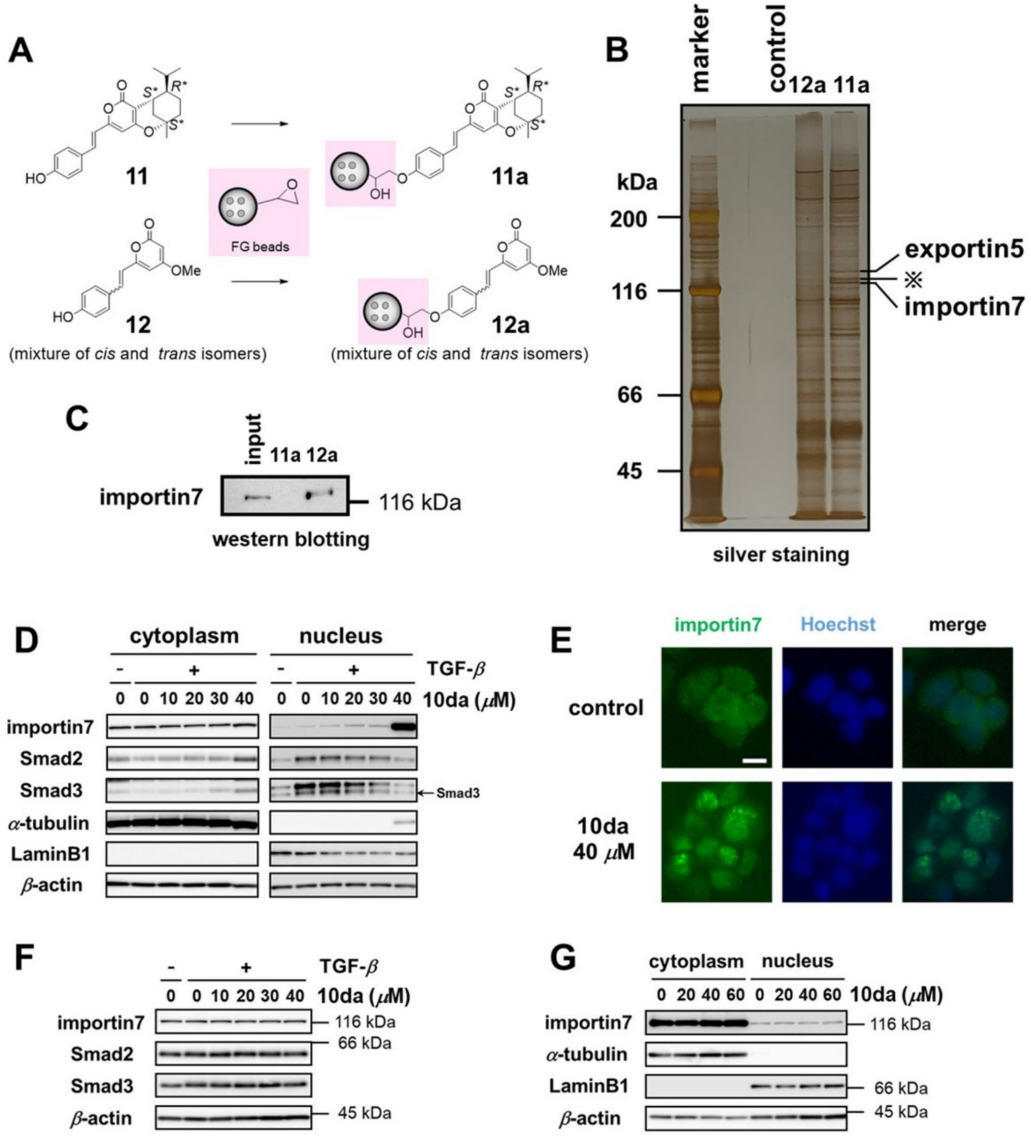
Identification of the target proteins of **10a**. **A** Scheme for the fixation of compounds **11** and **12** onto magnetic FG beads with epoxy linkers. **B** Binding proteins for **11a** and **12a** were purified from HT-29 cell extracts. The purified proteins were subjected to SDS-PAGE, followed by silver staining (B) or western blotting **C**. The input lane represents the 5% HT-29 cell extract used for the binding assay. ^※^We could not identify this protein in this study. (**D**, **F**, and **G**) HT-29 cells were treated with **10a** at the indicated concentrations. After incubation for 23 h, the cells were treated with ( +) or without ( −) TGF-*β* (10 ng/mL) in the presence of **10a** for 1 h. Importin7, Smad2, and Smad3 proteins were detected by western blotting. (G) Cells were fixed and stained with an anti-importin7 antibody. Nuclei were stained with Hoechst33342. In all Figures, scale bar = 10 mm

To clarify the mechanism by which **10da** inhibits Wnt/*β*-catenin signaling, we aimed to identify potential **10da** target proteins. To purify the **1da**-binding proteins, we used magnetic FG beads, which is a convenient method for the identification of bioactive compound-binding proteins. When using this approach, negative control experiments are important to distinguish target proteins from non-specific or irrelevant binding proteins. In the study described above, we determined that both the pyrone and monoterpene moieties on **1da** were necessary for Wnt/*β*-catenin pathway inhibition. Based on this structure–activity relationship, we designed and synthesized two compounds, one containing both moieties (**11**: active compound, growth inhibition at 50 mM: 74.50 ± 0.38%) and one only the pyrone moiety (**12**: inactive compound, IC_50_ > 100 mM) (Fig. 4A). These two compounds were conjugated to FG beads in *N*, *N*-dimethylformamide (DMF) and used for affinity purification. This approach enabled the discrimination of anticipated target proteins from non-specific binding proteins by comparing each binding protein. The **11**- or **12**-conjugated beads (**11a** and **12a**) were incubated with HT-29 whole-cell extracts. After washing with binding buffer, the bound proteins were eluted. The eluates were subjected to sodium dodecyl sulfate–polyacrylamide gel electrophoresis (SDS-PAGE) and silver staining. The three bands were excised and subjected to mass spectrometry. Exportin5 and importin7 were detected in cell extracts treated with **11a** but not in those treated with the control or **12a** (Fig. 4B). In addition, binding of **11a** to importin7 was confirmed by western blotting (Fig. 4C). These results suggested that **1da** might inhibit exportin5 and importin7 function by directly binding them. Aberrant exportin5 expression is associated with worse clinicopathological features and poor survival in patients with colorectal cancer [46]. Importantly, importin7 is overexpressed in several cancer cells such as cervical cancer cells and it contributes to tumor growth and a poor prognosis [47, 48]. In addition, it drives the nuclear import of Smad2, Smad3, and Smad4 [49], which enhances the transcriptional activity of TCF/LEF-specific reporters [50, 51]. Therefore, we assumed that importin7 was a target protein of **10da** involved in the mechanism of its Wnt/*β*-catenin pathway inhibition [25].

### Evaluation of importin7 functional inhibition in HT-29 cells by 10da

Wnt/*β*-catenin signaling pathway activation by Smad proteins occurs by their nuclear translocation followed by transforming growth factor (TGF-*β*) signaling pathway activation [52]. TGF-*β*1 activates the TGF-*β*/Smads signaling pathway by dimerizing type I and type II receptors, and this heterodimer phosphorylates Smad2 and Smad3 [51]. These phosphorylated proteins form complexes with Smad4, translocate to the nucleus, and interact with other transcription factors [53]. Thus, we speculated that **10da** may bind to importin7 and suppress nuclear translocation of Smad proteins. Therefore, we examined whether **10da** affected the quantities of Smad proteins in cytoplasmic and nuclear fractions under TGF-*β* stimulation using western blotting. Treatment with **10da** reduced Smad2 and Smad3 levels in the nuclear fraction in a dose-dependent manner, whereas the levels in the cytoplasmic fraction increased (Fig. 4D). However, Smad4 was detected in neither fraction. These results suggested that **10da** inhibited the nuclear translocation of Smad2 and Smad3. Interestingly, **1da** induced the nuclear accumulation of importin7 (Fig. 4D, 4E) without enhancing its expression (Fig. 4F). Nuclear importin7 accumulation was not observed after ICG-001 treatment (Fig. 4G). According to this result, we deduced **10da** inhibited the recycling process of importin7 or enhanced transition of importin7 to nucleus because importin7 was accumulated by **10da** treatment on HT-29 cells. Several inhibitors of nuclear transport factors, such as importin-*α* and importin-*β*, have been previously reported [54–56]. To the best of our knowledge, this is the first report of a compound with an inhibitory effect on importin7 function [25].

### Evaluation of the relationship between importin7 and the Wnt/*β*-catenin pathway

**Fig. 5:**
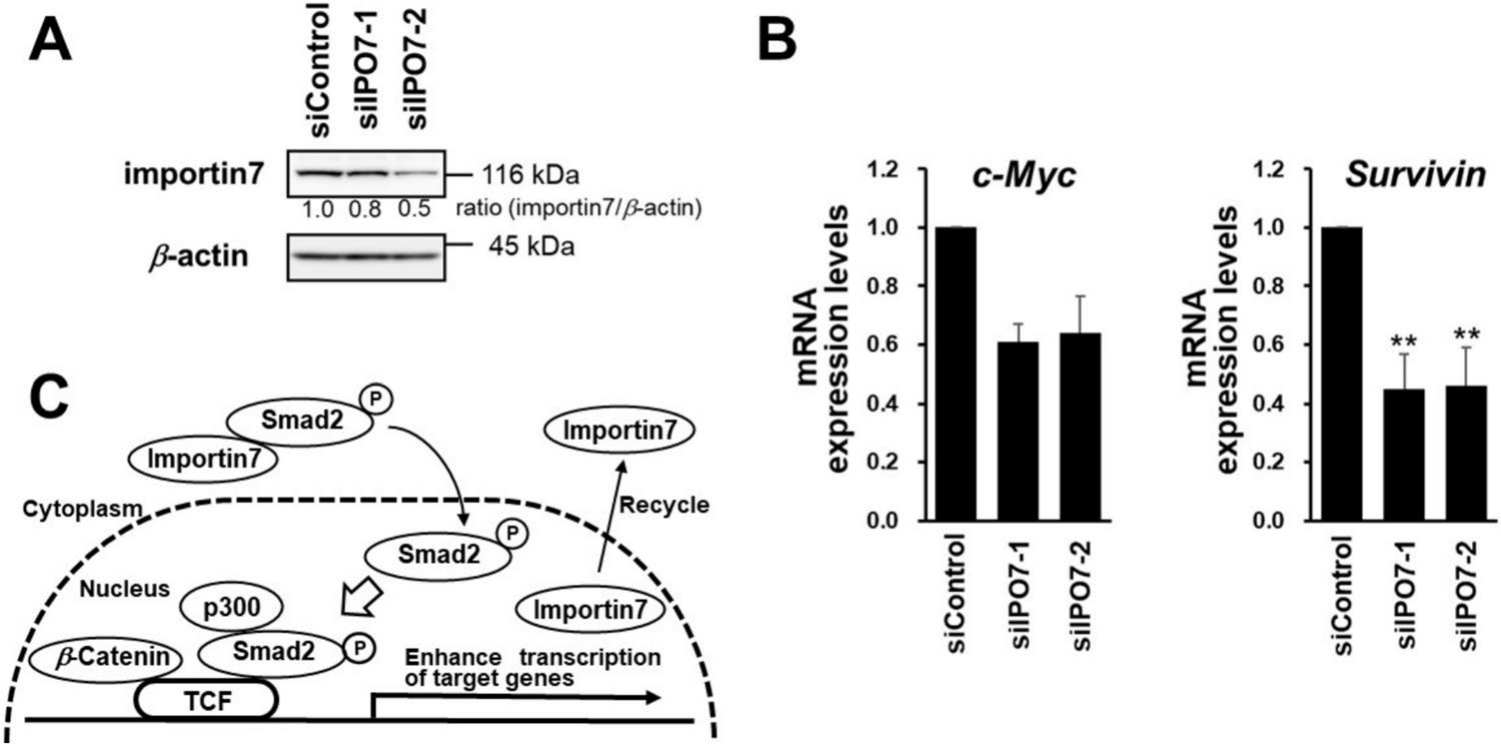
Knockdown of importin7 downregulates the expression of the target genes of the Wnt/*β*-catenin signaling pathway. **A, B** Non-target siRNA (siControl) and two independent importin7 siRNA (siIPO7-1 and siIPO7-2) were transfected into HT-29 cells. **A** Importin7 protein was detected using western blotting. The relative expression levels of importin7/*β*-actin compared to those of the control were shown. **B** RT-qPCR was performed to determine the mRNA levels for c-Myc and Survivin genes. The level of *β*-actin mRNA expression was used to normalize the data. The data are presented as mean ± SD of three independent experiments (**P < 0.01 compared with siControl). **C** A model of the crosstalk between the Wnt/β-catenin pathway and the Smad pathway together with the role of importin7

Several studies have reported the effect of importin7 cargos, such as Smad2, Smad3, and Smad4, on the Wnt/*β*-catenin signaling pathway [49, 52]. In addition, previous report suggests that *β*-catenin and Smad2 synergistically enhance the transcriptional activity of the Wnt/β-catenin target genes and Smad4 negatively affects this synergistic enhancement [50]. The amount of Smad4 is lower in HT-29 [57], and we could not detect Smad4 in HT-29 cells by western blotting; therefore, we discussed **10da** inhibits Wnt/*β*-catenin signaling pathway via reducing the amount of Smad2 in nucleus. However, the relationship between importin7 inhibition and Wnt/*β*-catenin signaling remains to be elucidated. Therefore, we investigated whether importin7 contributed to Wnt/*β*-catenin pathway activation. To investigate whether importin7 is involved in Wnt/*β*-catenin pathway activation, we knocked down importin7 in HT-29 cells and examined the expression of target genes of the pathway. Importin7 knockdown reduced c-myc and survivin mRNA expression relative to that in control cells (Fig. 5A, 5B). These results suggested that importin7 contributes to Wnt/*β*-catenin pathway activation via reducing the amount of importin7 cargo proteins such as Smad2 in nucleus. Also, this result may suggest that Smad2 has an important role in the transcriptional activity of the Wnt/β-catenin pathway as previous report [50] (Fig. 5C). Therefore, we concluded that importin7 should be a target protein of **10da** for the inhibition of the Wnt/*β*-catenin signaling pathway. In conclusion, **10da** may represent a novel cancer treatment agent capable of killing drug-resistant cancer cells such as CSCs [25].

## Conclusion

We identified several bioactive, naturally occurring compounds that inhibit the function or expression of chemotherapy resistance factors in cancer cells. These compounds may be novel cancer treatment agents that inhibit HSP expression. In the course of these studies, we established new screening methods, such as 24 h time-lapse imaging analysis and a *luc* assay system using pGL105/C3H cells. The imaging analysis revealed cell death induction in HeLa cells treated with low ADR concentrations (1.0 μg/ml). Because this analysis detected cell death, it allowed identification of chemotherapy resistance factor inhibitors, such as HSP expression inhibitors, that do not strongly inhibit cell proliferation. In addition, a *luc* assay system using pGL105/C3H cells enabled the efficient identification of HSP105 expression inhibitors. Moreover, the compounds discovered using these assay systems showed inhibitory effects on HSP105 expression in HeLa cells. These assays provide a basis for the development of novel cancer treatment agents that target chemotherapy resistance factors.

Next, I introduced linderapyrone (**10**), isolated from *L. umbellata*, a novel inhibitor of Wnt/*β*-catenin signaling. In a structure–activity relationship study of **10**, we succeeded in synthesizing a stronger inhibitor, **10da**. In addition, we confirmed the inhibitory effect of **10da** against Wnt/*β*-catenin signaling in HT-29 cell using western blotting analysis. Further, we investigated the expression levels of *β*-catenin, c-myc, and survivin after **10da** treatment. Importantly, we identified the target protein of **10da** as importin7 via an affinity assay using magnetic FG beads. Compound **10da** is the first reported inhibitor of importin7. In this experiment, both bioactive and inactive compounds were used to exclude non-specific binding proteins. Finally, importin7 knockdown reduced c-myc and survivin mRNA expression relative to that in control cells. Thus, **10da** may be an important seed compound for killing chemotherapy-resistant cancer cells such as CSCs by inhibiting importin7 function strongly related to the Wnt/*β*-catenin signaling pathway.

## References

[1] Yamane T, Saito Y, Teshima H, Hagino M, Kakihana A, Sato S, Shimada M, Kato Y, Kuga T, Yamagishi N, Nakayama Y (2019) Hsp105 suppresses adriamycin-induced cell death via nuclear localization signal-dependent nuclear accumulation. J Cell Biochem 120:17951–17962. 10.1002/jcb.29062 31173393 10.1002/jcb.29062

[2] Dolatabadi S, Jonasson E, Lindén M, Fereydouni B, Bäcksten K, Nilsson M, Martner A, Forootan A, Fagman H, Landberg G, Åman P, Ståhlberg A (2019) JAK-STAT signalling controls cancer stem cell properties including chemotherapy resistance in myxoid liposarcoma. Int J Cancer 145:435–449. 10.1002/ijc.32123 30650179 10.1002/ijc.32123 PMC6590236

[3] Testa U, Pelosi E, Castelli G (2020) Cancer stem cell targeted therapies. Ann Ist Super Sanita 56:336–350. 10.4415/ANN_20_03_12 32959800 10.4415/ANN_20_03_12

[4] Sethy C, Kundu CN (2021) 5-Fluorouracil (5-FU) resistance and the new strategy to enhance the sensitivity against cancer: implication of DNA repair inhibition. Biomed Pharmacother 137:111285. 10.1016/j.biopha.2021.111285 33485118 10.1016/j.biopha.2021.111285

[5] Chen S, Zhao Y, Liu S, Zhang J, Assaraf YG, Cui W, Wang L (2022) Epigenetic enzyme mutations as mediators of anti-cancer drug resistance. Drug Resist Updat 61:100821. 10.1016/j.drup.2022.100821 35219075 10.1016/j.drup.2022.100821

[6] Oberli-Schrämmli AE, Joncourt F, Stadler M, Altermatt HJ, Buser K, Ris HB, Schmid U, Cerny T (1994) Parallel assessment of glutathione-based detoxifying enzymes, O6-alkylguanine-DNA alkyltransferase and P-glycoprotein as indicators of drug resistance in tumor and normal lung of patients with lung cancer. Int J Cancer 59:629–636. 10.1002/ijc.2910590509 7960235 10.1002/ijc.2910590509

[7] Szakács G, Paterson JK, Ludwig JA, Booth-Genthe C, Gottesman MM (2006) Targeting multidrug resistance in cancer. Nat Rev Drug Discov 5:219–234. 10.1038/nrd1984 16518375 10.1038/nrd1984

[8] Damiani RM, Moura DJ, Viau CM, Caceres RA, Henriques JAP, Saffi J (2016) Pathways of cardiac toxicity: Comparison between chemotherapeutic drugs doxorubicin and mitoxantrone. Arch Toxicol 90:2063–2076. 10.1007/s00204-016-1759-y 27342245 10.1007/s00204-016-1759-y

[9] Cheung KG, Cole LK, Xiang B, Chen K, Ma X, Myal Y, Hatch GM, Tong Q, Dolinsky VW (2015) Sirtuin-3 (SIRT3) protein attenuates doxorubicin-induced oxidative stress and improves mitochondrial respiration in H9c2 cardiomyocytes. J Biol Chem 290:10981–10993. 10.1074/jbc.M114.607960 25759382 10.1074/jbc.M114.607960 PMC4409259

[10] Yun CW, Kim HJ, Lim JH, Lee SH (2019) Heat shock proteins: agents of cancer development and therapeutic targets in anti-cancer therapy. Cells 9:60. 10.3390/cells9010060 31878360 10.3390/cells9010060 PMC7017199

[11] Singh SK, Clarke ID, Terasaki M, Bonn VE, Hawkins C, Squire J, Dirks PB (2003) Identification of a cancer stem cell in human brain tumors. Cancer Res 15:5821–5828 14522905

[12] Hemmati HD, Nakano I, Lazareff JA, Masterman-Smith M, Geschwind DH, Bronner-Fraser M, Kornblum HI (2003) Cancerous stem cells can arise from pediatric brain tumors. Proc Natl Acad Sci U S A 100:15178–15183. 10.1073/pnas.2036535100 14645703 10.1073/pnas.2036535100 PMC299944

[13] Matsumoto T, Kitagawa T, Imahori D, Yoshikawa H, Okayama M, Kobayashi M, Kojima N, Yamashita M, Watanabe T (2021) Cell death inducing activities via Hsp inhibition of the sesquiterpenes isolated from *Valeriana fauriei*. J Nat Med 75:942–948. 10.1007/s11418-021-01543-9 34212302 10.1007/s11418-021-01543-9

[14] Matsumoto T, Imahori D, Ohnishi E, Okayama M, Kitagawa T, Ohta T, Yoshida T, Kojima N, Yamashita M, Watanabe T (2022) Chemical structures and induction of cell death via heat shock protein inhibition of the prenylated phloroglucinol derivatives isolated from *Hypericum erectum*. Fitoterapia 156:105097. 10.1016/j.fitote.2021.105097 34890752 10.1016/j.fitote.2021.105097

[15] Matsumoto T, Ohnishi E, Kitagawa T, Okayama M, Saito Y, Yoshikawa H, Ohta T, Yoshida T, Nakayama Y, Watanabe T (2023) Azaphilones produced by *Penicillium maximae* with their cell death-inducing activity on Adriamycin-treated cancer cell. Genes Environ 45:5. 10.1186/s41021-023-00261-w 36658662 10.1186/s41021-023-00261-w PMC9850696

[16] Matsumoto T, Imahori D, Saito Y, Zhang W, Ohta T, Yoshida T, Nakayama Y, Ashihara E, Watanabe T (2020) Cytotoxic activities of sesquiterpenoids from the aerial parts of *Petasites japonicus* against cancer stem cells. J Nat Med 74:689–701. 10.1007/s11418-020-01420-x 32535872 10.1007/s11418-020-01420-x

[17] Matsumoto T, Kitagawa T, Imahori D, Saito Y, Ohta T, Yoshida T, Nakayama Y, Watanabe T (2021) Linderapyrone; a Wnt signal inhibitor isolated from *Lindera umbellata*. Bioorg Med Chem Lett 45:128161. 10.1016/j.bmcl.2021.128161 34062253 10.1016/j.bmcl.2021.128161

[18] Yoneda T, Kojima N, Matsumoto T, Imahori D, Ohta T, Yoshida T, Watanabe T, Matsuda H, Nakamura S (2022) Construction of sulfur-containing compounds with anticancer stem cell activity using thioacrolein derived from garlic based on nature-inspired scaffolds. Org Biomol Chem 20:196. 10.1039/D1OB01992A 10.1039/d1ob01992a 34878480

[19] Yoshikawa H, Matsumoto T, Kitagawa T, Okayama M, Ohta T, Yoshida T, Watanabe T (2022) Anti-proliferative effects of iridoids from *Valeriana fauriei* on cancer stem cells. Int J Mol Sci 23:14206. 10.3390/ijms232214206 36430685 10.3390/ijms232214206 PMC9698980

[20] Nakamura S, Sugimoto S, Yoneda T, Shinozaki A, Yoshiji M, Matsumoto T, Nakashima S, Matsuda H (2023) Antiproliferative activities of diterpenes from leaves of *Isodon trichocarpus* against cancer stem cells. Chem Pharm Bull 46:502–507. 10.1248/cpb.c22-00914 10.1248/cpb.c22-00914 37394598

[21] Ugawa K, Nakao M, Sawada C, Matsumoto T, Kitagawa T, Ohki Y, Araki K, Nakamura S (2023) One-pot synthesis of carbazoles by a domino reaction using microwave heating and antiproliferative activities of constituents from *Murraya* plants against cancer stem cells. Heterocycles 106:725–733

[22] Matsumoto T, Yoshikawa H, Kitagawa T, Imahori D, Ohta T, Yoshida T, Watanabe T (2023) Chemical structures and anti-proliferative effects of *Valeriana fauriei* constituents on cancer stem cells. Chem Pharm Bull 71:495–501. 10.1248/cpb.c21-00832 10.1248/cpb.c21-00832 37394597

[23] Okayama M, Matsumoto T, Kitagawa T, Nakamura S, Ohta T, Yoshida T, Watanabe T (2024) Cytotoxic activities of alkaloid constituents from the climbing stems and rhizomes of *Sinomenium acutum* against cancer stem cells. J Nat Med 78:226–235. 10.1007/s11418-023-01744-4 37656375 10.1007/s11418-023-01744-4

[24] Araki K, Hara M, Hamada S, Matsumoto T, Nakamura S (2024) Antiproliferative activities of cynaropicrin and related compounds against cancer stem cells. Chem Pharm Bull 72:200–208. 10.1248/cpb.c23-00811 10.1248/cpb.c23-00811 38382968

[25] Kitagawa T, Matsumoto T, Ohta T, Yoshida T, Saito Y, Nakayama Y, Hadate Y, Ashihara E, Watanabe T (2024) Linderapyrone analogue LPD-01 as a cancer treatment agent by targeting importin7. J Nat Med 78:370–381. 10.1007/s11418-023-01774-y 38265612 10.1007/s11418-023-01774-y

[26] Kitagawa T, Matsumoto T, Imahori D, Kobayashi M, Okayama M, Ohta T, Yoshida T, Watanabe T (2021) Limonoids isolated from the *Fortunella crassifolia* and the *Citrus junos* with their cell death inducing activity on Adriamycin treated cancer cell. J Nat Med 75:998–1004. 10.1007/s11418-021-01528-8 33991286 10.1007/s11418-021-01528-8

[27] Matsumoto T, Imahori D, Achiwa K, Saito Y, Ohta T, Yoshida T, Kojima N, Yamashita M, Nakayama Y, Watanabe T (2020) Chemical structures and cytotoxic activities of the constituents isolated from *Hibiscus tiliaceus*. Fitoterapia 142:104524. 10.1016/j.fitote.2020.104524 32092530 10.1016/j.fitote.2020.104524

[28] Whitesell L, Mimnaugh EG, De Costa B, Myers CE, Neckers LM (1994) Inhibition of heat shock protein HSP90-pp60v-src heteroprotein complex formation by benzoquinone ansamycins: essential role for stress proteins in oncogenic transformation. Proc Natl Acad Sci USA 91:8324–8328. 10.1073/pnas.91.18.8324 8078881 10.1073/pnas.91.18.8324 PMC44598

[29] Uno T, Kawai Y, Yamashita S et al (2019) Discovery of 3-ethyl-4-(3-isopropyl-4-(4-(1-methyl-1*H*-pyrazol-4-yl)-1*H*-imidazol-1-yl)-1*H*-pyrazolo[3,4-b]pyridin-1-yl)benzamide (Tas-116) as a potent, selective, and orally available HSP90 inhibitor. J Med Chem 62:531–540. 10.1021/acs.jmedchem.8b01085 30525599 10.1021/acs.jmedchem.8b01085

[30] Tatokoro M, Koga F, Yoshida S, Kihara K (2015) Heat shock protein 90 targeting therapy: state of the art and future perspective. EXCLI J 14:48–58 26600741 10.17179/excli2014-586 PMC4652636

[31] Sõti C, Nagy E, Giricz Z, Vígh L, Csermely P, Ferdinandy P (2005) Heat shock proteins as emerging therapeutic targets. Br J Pharmacol 146:769–780. 10.1038/sj.bjp.0706396 16170327 10.1038/sj.bjp.0706396 PMC1751210

[32] Griffin TM, Valdez TV, Mestril R (2003) Radicicol activates heat shock protein expression and cardioprotection in neonatal rat cardiomyocytes. Am J Heart Circ Physiol 287:H1081–H1088. 10.1152/ajpheart.00921.2003 10.1152/ajpheart.00921.2003 15117720

[33] Cockburn IL, Pesce ER, Pryzborski JM, Davies-Coleman MT, Clark PG, Keyzers RA, Stephens LL, Blatch GL (2011) Screening for small molecule modulators of Hsp70 chaperone activity using protein aggregation suppression assays: inhibition of the plasmodial chaperone PfHsp70-1. Biol Chem 392:431–438. 10.1515/BC.2011.040 21426241 10.1515/BC.2011.040

[34] Ishihara K, Horiguchi K, Yamagishi N, Hatayama T (2003) Identification of sodium salicylate as an hsp inducer using a simple screening system for stress response modulators in mammalian cells. Eur J Biochem 270:3461–3468. 10.1046/j.1432-1033.2003.03740.x 12899704 10.1046/j.1432-1033.2003.03740.x

[35] Yoon YJ, Kim JA, Shin KD, Shin DS, Han YM, Lee YJ, Lee JS, Kwon BM, Han DC (2011) KRIBB11 inhibits HSP70 synthesis through inhibition of heat shock factor 1 function by impairing the recruitment of positive transcription elongation factor b to the hsp70 promoter. J Biol Chem 286:1737–1747. 10.1074/jbc.M110.179440 21078672 10.1074/jbc.M110.179440 PMC3023468

[36] Sarabia-Sánchez MA, Moreno-Londoño AP, Castañeda-Patlán MC, Alvarado-Ortiz E, Martínez-Morales JC, Robles-Flores M (2023) Non-canonical Wnt/Ca2⁺ signaling is essential to promote self-renewal and proliferation in colon cancer stem cells. Front Oncol 13:1121787. 10.3389/fonc.2023.1121787 36969011 10.3389/fonc.2023.1121787 PMC10036746

[37] Yang L, Shi P, Zhao G, Xu J, Peng W, Zhang J, Zhang G, Wang X, Dong Z, Chen F, Cui H (2020) Targeting cancer stem cell pathways for cancer therapy. Signal Transduct Target Ther 5:8. 10.1038/s41392-020-0110-5 32296030 10.1038/s41392-020-0110-5 PMC7005297

[38] Cheng H, Cenciarelli C, Nelkin G, Tsan R, Fan D, Cheng-Mayer C, Fidler IJ (2005) Molecular mechanism of hTid-1, the human homolog of *Drosophila* tumor suppressor l(2)Tid, in the regulation of NF-kappaB activity and suppression of tumor growth. Mol Cell Biol 25:44–59. 10.1128/MCB.25.1.44-59.2005 15601829 10.1128/MCB.25.1.44-59.2005 PMC538758

[39] Chen HW, Lee JY, Huang JY, Wang CC, Chen WJ, Su SF, Huang CW, Ho CC, Chen JJ, Tsai MF, Yu SL, Yang PC (2008) Curcumin inhibits lung cancer cell invasion and metastasis through the tumor suppressor HLJ1. Cancer Res 68:7428–7438. 10.1158/0008-5472.CAN-07-6734 18794131 10.1158/0008-5472.CAN-07-6734

[40] Lv LH, Wan YL, Lin Y, Zhang W, Yang M, Li GL, Lin HM, Shang CZ, Chen YJ, Min J (2012) Anticancer drugs cause release of exosomes with heat shock proteins from human hepatocellular carcinoma cells that elicit effective natural killer cell antitumor responses *in vitro*. J Biol Chem 287:15874–15885. 10.1074/jbc.M112.340588 22396543 10.1074/jbc.M112.340588 PMC3346092

[41] Sato T, Arai M, Yixizhuoma, et al (2020) Cadinane sesquiterpenoids isolated from *Santalum album* using a screening program for Wnt signal inhibitory activity. J Nat Med 74:476–481 31863259 10.1007/s11418-019-01380-x

[42] Ishibashi M (2019) Screening for natural products that affect Wnt signaling activity. J Nat Med 73:697–705. 10.1007/s11418-019-01320-9 31147959 10.1007/s11418-019-01320-9 PMC6713684

[43] Wakabayashi R, Hattori Y, Hosogi S, Toda Y, Ashihara E (2020) A novel dipeptide type inhibitor of the Wnt/β-catenin pathway suppresses proliferation of acute myelogenous leukemia cells. Biochem Biophys Res Commun 535:73–79. 10.1016/j.bbrc.2020.12.027 33341676 10.1016/j.bbrc.2020.12.027

[44] Shono T, Ishikawa N, Toume K et al (2016) Cerasoidine, a bis-aporphine alkaloid isolated from *Polyalthia cerasoides* during screening for Wnt signal inhibitors. J Nat Prod 79:2083–2088. 10.1021/acs.jnatprod.6b00409 27490091 10.1021/acs.jnatprod.6b00409

[45] Bae ES, Kim YM, Kim DH et al (2020) Anti-proliferative activity of nodosin, a diterpenoid from *Isodon serra*, via regulation of Wnt/β-catenin signaling pathways in human colon cancer cells. Biomol Ther 28:465–472. 10.4062/biomolther.2020.003 10.4062/biomolther.2020.003 PMC7457175 32394670

[46] Shigeyasu K, Okugawa Y, Toden S, Boland CR, Goel A (2017) Exportin-5 functions as an oncogene and a potential therapeutic target in colorectal cancer. Clin Cancer Res 23:1312–1322. 10.1158/1078-0432.CCR-16-1023 27553833 10.1158/1078-0432.CCR-16-1023 PMC5435115

[47] Chen J, Hu Y, Teng Y, Yang BK (2021) Increased nuclear transporter importin 7 contributes to the tumor growth and correlates with CD8 T cell infiltration in cervical cancer. Front Cell Dev Biol 9:732786. 10.3389/fcell.2021.732786 34650978 10.3389/fcell.2021.732786 PMC8505702

[48] Cai GX, Kong WY, Liu Y, Zhong SY, Liu Q, Deng YF, Ye GL (2023) Nuclear transport maintenance of USP22-AR by Importin-7 promotes breast cancer progression. Cell Death Discov 9:211. 10.1038/s41420-023-01525-8 37391429 10.1038/s41420-023-01525-8 PMC10313651

[49] Xu L, Yao X, Chen X, Lu P, Zhang B, Ip YT (2007) Msk is required for nuclear import of TGF-β/BMP-activated Smads. J Cell Biol 178:981–994. 10.1083/jcb.200703106 17785517 10.1083/jcb.200703106 PMC2064622

[50] Hirota M, Watanabe K, Hamada S, Sun Y, Strizzi L, Mancino M, Nagaoka T, Gonzales M, Seno M, Bianco C, Salomon DS (2008) Smad2 functions as a co-activator of canonical Wnt/β-catenin signaling pathway independent of Smad4 through histone acetyltransferase activity of p300. Cell Signal 20:1632–1641. 10.1016/j.cellsig.2008.05.003 18595660 10.1016/j.cellsig.2008.05.003 PMC2578836

[51] Lei S, Dubeykovskiy A, Chakladar A, Wojtukiewicz L, Wang TC (2004) The murine gastrin promoter is synergistically activated by transforming growth factor-β/Smad and Wnt signaling pathways. J Biol Chem 279:42492–42502. 10.1074/jbc.M404025200 15292219 10.1074/jbc.M404025200

[52] Yao X, Chen X, Cottonham C, Xu L (2008) Preferential utilization of imp7/8 in nuclear import of Smads. J Biol Chem 283:22867–22874. 10.1074/jbc.M801320200 18519565 10.1074/jbc.M801320200 PMC2504884

[53] Tian H, Liu C, Yu J, Han J, Du J, Liang S, Wang W, Liu Q, Lian R, Zhu T, Wu S, Tao T, Ye Y, Zhao J, Yang Y, Zhu X, Cai J, Wu J, Li M (2023) PHF14 enhances DNA methylation of SMAD7 gene to promote TGF-β-driven lung adenocarcinoma metastasis. Cell Discov 9:41. 10.1038/s41421-023-00528-0 37072414 10.1038/s41421-023-00528-0 PMC10113255

[54] Soderholm JF, Bird SL, Kalab P, Sampathkumar Y, Hasegawa K, Uehara-Bingen M, Weis K, Heald R (2011) Importazole, a small molecule inhibitor of the transport receptor importin-β. ACS Chem Biol 6:700–708. 10.1021/cb2000296 21469738 10.1021/cb2000296 PMC3137676

[55] Martiniano B (2021) Elucidation of the inhibitory activity of ivermectin with host nuclear importin α and several SARS-CoV-2 targets. J Biomol Struct Dyn 40:8375–8383. 10.1080/07391102.2021.1911857 33843474 10.1080/07391102.2021.1911857 PMC8054936

[56] Van Der Watt PJ, Chi A, Stelma T, Stowell C, Strydom E, Carden S, Angus L, Hadley K, Lang D, Wei W, Birrer MJ, Trent JO, Leaner VD (2016) Targeting the nuclear import receptor Kpnβ1 as an anticancer therapeutic. Mol Cancer Ther 15:560–573. 10.1158/1535-7163.MCT-15-0052 26832790 10.1158/1535-7163.MCT-15-0052

[57] Refaat B, Zekri J, Aslam A, Ahmad J, Baghdadi MA, Meliti A, Idris S, Sultan S, Alardati H, Saimeh HA, Alsaegh A, Alhadrami M, Hamid T, Naeem ME, Elsamany SA (2021) Profiling activins and follistatin in colorectal cancer according to clinical stage, tumour sidedness and Smad4 status. Pathol Oncol Res 27:1610032. 10.3389/pore.2021.1610032 34867090 10.3389/pore.2021.1610032 PMC8634429

